# Novel In-Frame Deletion in *HTRA1* Gene, Responsible for Stroke at a Young Age and Dementia—A Case Study

**DOI:** 10.3390/genes12121955

**Published:** 2021-12-07

**Authors:** Julija Grigaitė, Kamilė Šiaurytė, Eglė Audronytė, Eglė Preikšaitienė, Birutė Burnytė, Erinija Pranckevičienė, Aleksandra Ekkert, Algirdas Utkus, Dalius Jatužis

**Affiliations:** 1 Center of Neurology, Faculty of Medicine, Vilnius University, 03101 Vilnius, Lithuania; julgrigaite@gmail.com (J.G.); egle.audronyte@mf.vu.lt (E.A.); sacha.gavrilova@gmail.com (A.E.); 2 Center for Medical Genetics, Faculty of Medicine, Vilnius University, 03101 Vilnius, Lithuania; kamilesiaur@gmail.com (K.Š.); egle.preiksaitiene@mf.vu.lt (E.P.); birute.burnyte@mf.vu.lt (B.B.); erinija.pranckeviciene@mf.vu.lt (E.P.); algirdas.utkus@mf.vu.lt (A.U.); 3 Department of Systems Analysis, Faculty of Informatics, Vytautas Magnus University, 44404 Kaunas, Lithuania

**Keywords:** HTRA1, heterozygous HTRA1 gene mutation, cerebral small vessel disease, CARASIL, next generation sequencing

## Abstract

Biallelic mutations in the high-temperature requirement A serine peptidase 1 (HTRA1) gene are known to cause an extremely rare cerebral autosomal recessive arteriopathy with subcortical infarcts and leukoencephalopathy (CARASIL), which belongs to the group of hereditary cerebral small vessel diseases and is mainly observed in the Japanese population. Even though this pathology is inherited in an autosomal recessive manner, recent studies have described symptomatic carriers with heterozygous HTRA1 mutations who have milder symptoms than patients with biallelic HTRA1 mutations. We present the case of a Lithuanian male patient who had a stroke at the age of 36, experienced several transient ischemic attacks, and developed an early onset, progressing dementia. These clinical symptoms were associated with extensive leukoencephalopathy, lacunar infarcts, and microbleeds based on brain magnetic resonance imaging (MRI). A novel heterozygous in-frame HTRA1 gene deletion (NM_002775.5:c.533_535del; NP_002766.1:p.(Lys178del)) was identified by next generation sequencing. The variant was consistent with the patient’s phenotype, which could not be explained by alternative causes, appeared highly deleterious after in silico analysis, and was not reported in the medical literature or population databases to date.

## 1. Introduction

Cerebral small vessel disease (CSVD) is a group of disorders that affect the small vessels of the brain and usually manifest as a stroke and cognitive dysfunction [1]. There is a broad spectrum of etiological factors and pathological processes underlying CSVD, but it could be broadly classified as either sporadic or congenital. Monogenic forms are less prevalent, accounting for up to 5% of all SVD, and they are mostly inherited in an autosomal dominant manner [2]. Cerebral autosomal dominant arteriopathy with subcortical infarcts and leukoencephalopathy (CADASIL) is the most common inherited CSVD, determined by the mutations in the *NOTCH3* gene [3]. As the eponym suggests, CARASIL (cerebral autosomal recessive arteriopathy with subcortical infarcts and leukoencephalopathy) is similar to CADASIL from a clinical and neuroradiological point of view, although it is inherited in an autosomal recessive manner and caused by the mutations in the high-temperature requirement A serine peptidase 1 (*HTRA1*) gene. CARASIL is extremely rare and mostly observed in the Japanese population [4,5].

CARASIL was originally described as CSVD that is caused by biallelic mutation specifically [4]. However, recent studies have shown that heterozygous variants are not necessarily asymptomatic: some neuroradiological and clinical findings demonstrate a possible autosomal dominant pattern of inheritance [6,7,8]. Thus, there are two possible phenotypes related to *HTRA1* gene mutation: “classical” CARASIL syndrome, inherited in an autosomal recessive manner (MIM 600142), and cerebral autosomal dominant arteriopathy with subcortical infarcts and leukoencephalopathy, type 2 (CADASIL2) or, alternatively, HTRA1-related cerebral small vessel disease (HTRA1-CSVD) (MIM 616779) [6,9]. Both are similar in their presentation, although the latter is generally milder [6]. They are characterized by acute cerebral ischemic events at a young age and progressive dementia, together with gait disturbance and mood changes. CARASIL typically manifests at a younger age and has a more progressive course. Scalp alopecia, lumbago associated with spondylosis deformans, urinary incontinence, and pseudobulbar palsy are linked to CARASIL as well and rarely seen in HTRA1-CSVD. It is worth mentioning that cardiovascular risk factors affect the time and severity of clinical manifestation in patients with heterozygous *HTRA1* pathogenic variants [6,10,11]. Neuroimaging features (severe leukoencephalopathy, symmetrical subcortical, and paraventricular white matter hyperintensities with relative sparing of U fibers, lacunar infarctions, and microbleeds) are seen in both CARASIL and HTRA1-CSVD patients, although they tend to be less obvious in the latter population [6,11,12].

As there are only a little more than 50 symptomatic carriers of *HTRA1* pathogenic variants and around 20 mutations distinguished in the literature, the clinical picture of HTRA1-CSVD is barely understood, and a possible genotype–phenotype correlation is still unclear [12]. To expand this knowledge, the aim of this paper is to present the case of a patient with severe symptoms of dementia and stroke at a young age related to a novel heterozygous in-frame HTRA1 gene deletion.

## 2. Materials and Methods

### 2.1. Case Report

**Figure 1:**
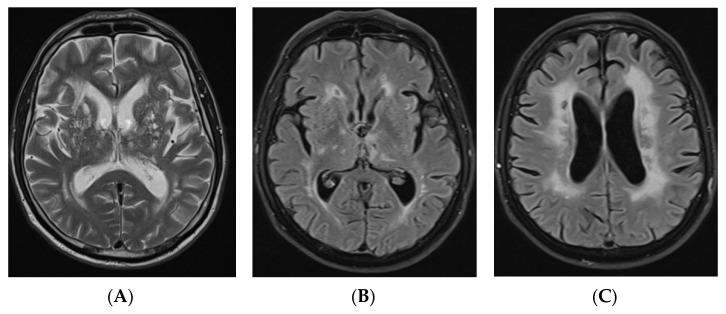
Axial T2-weighted image (**A**) and FLAIR axial images (**B**,**C**) of brain MRI demonstrating subcortical and periventricular T2/T2 dark-fluid hyperintensities, Fazekas scale score 2–3, lacunar lesions in the thalamus bilaterally, and hydrocephalus (HI—66). FLAIR: fluid attenuated inversion recovery; MRI: magnetic resonance imaging, HI: Huckman index.

A 59-year-old Lithuanian male presented to our department due to deterioration of cognitive functions that had been observed for 2–3 years and gotten worse over the past three days. The patient could not perform some simple tasks in everyday life and lost his previous interests. He maintained some independence though, such as being able to go to the supermarket and do housework unsupervised. Past medical history was significant for dyslipidaemia, arterial hypertension, and stroke at the age of 36 with mild right hemiparesis. He also experienced several episodes of aphasia, which could be considered as transient ischemic attacks (TIAs). The patient had a history of smoking for a long time. He was born full-term and healthy; his parents, four siblings, and two offspring did not have any relevant health problems and no hereditary diseases were identified among family members. On neurological examination, mild bilateral dysmetria was observed and the mental examination revealed executive dysfunction and pronounced cognitive slowing. Mini–Mental State Examination (MMSE) score was 25, Frontal Assessment Battery (FAB) score was 5, phonemic fluency (words beginning with P) was 4 in one minute, and semantic fluency (animals) was 3 in one minute. Laboratory blood tests revealed significant dyslipidaemia (total cholesterol level—7.55 mmol/L, low-density lipoprotein level—5.82 mmol/L). Cerebrospinal fluid analysis was unremarkable. Low grade bilateral internal and external carotid artery stenosis was detected on carotid ultrasound. Brain magnetic resonance imaging (MRI) revealed communicating hydrocephalus, most likely due to brain atrophy and secondary brain changes, with no obvious cause of obstruction in the ventricles (Huckman index was equal to 66; the width of the third ventricle was equal to 10 mm), and extensive leukoencephalopathy, Fazekas scale score 2–3, lacunar lesions in the dorsal part of pons, thalamus bilaterally, and right cerebellar hemisphere (Figure 1).

**Figure 2:**
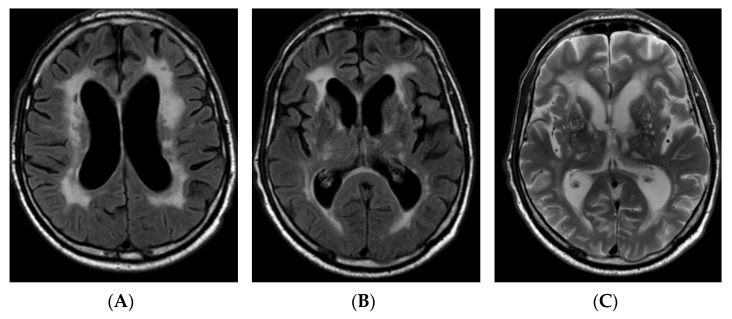
FLAIR axial images (**A**,**B**) and axial T2-weighted image (**C**) of brain MRI, performed after 3 years of initial brain MRI, demonstrating more subcortical and periventricular T2/T2 dark-fluid hyperintensities, Fazekas scale score 3, new lacunar lesions in right thalamus, progressive ventricular dilation (HI—82). FLAIR: fluid attenuated inversion recovery; MRI: magnetic resonance imaging; HI: Huckman index.

Since the patient developed early onset progressive dementia, had a stroke at a young age, several TIAs, and brain MRI was significant for extensive leukoencephalopathy, genetic testing by next generation sequencing for inherited cerebral small vessel disease was performed. On follow-up, the patient began manifesting positive psychiatric symptoms (hallucinations, delusions, anxiety) at the age of 60 that required several hospitalisations to the psychiatric ward. Cognitive functions further deteriorated from baseline MMSE score of 25 to 14 in 3 years, and the patient gradually became fully dependent in daily life. He also developed bladder and bowel incontinence and gait apraxia at the age of 62. In parallel, brain MRI showed evolution of findings: communicating hydrocephalus and leukoencephalopathy were progressing over time (HI was equal to 82, the width of the third ventricle was equal to 11 mm, Fazekas scale score 3), and new lacunar ischemic lesions and hemosiderin deposits appeared (Figure 2).

### 2.2. Gene Sequencing

Next generation sequencing (NGS) was performed on genomic DNA using Human Core Exome Kit (Twist Bioscience). The 33 Mb exome region covering 21,534 genes from GRCh38.p13 primary assembly [13] was analyzed, except for 1394 genes, which were not included in target region. Genomic libraries were prepared, and sequencing was performed in CeGaT Sequencing Service Centre, Tübingen, Germany. Demultiplexed FASTQ data were obtained and sent to Vilnius University Hospital Santaros Klinikos, Vilnius, Lithuania for analysis. Reads were aligned to GRCh38 human genome reference sequence by BWA MEM (Burrows–Wheeler Alignment with Maximal Exact Matches) v0.7.17 algorithm. Data were visualized using IGV (the Integrative Genomics Viewer) [14] tool. GATK v.3.8 algorithm was used for variant calling, which were further annotated and filtered by their functional impact (SIFT and PolyPhen-2 scores, MutationTaster), genomic databases (ClinVar [15], OMIM [16]), allelic frequency in population (1000 Genome Project [17], ExAC, gnomAD databases [18]), biomedical literature sources (Pubmed [19]) employing Ensembl VEP [20], Vcfanno software [21]. HGVS nomenclature [22] was used to annotate. ABCC6, APP, ATP1A2, CACNA1A, COL3A1, COL4A1, COL4A2, COLGALT1, FOXC1, GLA, HTRA1, NOTCH3, TREX1 genes, associated with familial cerebral small vessel disease, have been analyzed. Variants were classified following the guidelines of the American College of Medical Genetics and Genomics (ACMG [23]). Only variants that passed quality and coverage filters and showed >99.9% detection reliability were analyzed.

## 3. Results

The next generation sequencing identified a novel heterozygous in-frame HTRA1 gene deletion (NM_002775.5:c.533_535del; NP_002766.1:p.(Lys178del)) in the highly conserved region of exon 2, located in the linker domain of the protein. The variant had not been previously reported in the scientific literature or the Human Gene Mutation Database (HGMD) and was not found in reference population databases gnomAD, ExAC, or 1000 Genome Project. The deletion is not in the repeat region of the HTRA1 gene. It was predicted in silico to potentially alter the protein features; the MutationTaster score was 0.99. However, there are no documented functional studies to prove this. Since the disorders related to the HTRA1 phenotype are very specific and concordant with the patient’s phenotype, the variant has been classified as likely pathogenic.

## 4. Discussion

The HTRA1 gene is located on chromosome 10 (10q26) and encodes high temperature requirement serine protease. The protein consists of several parts that include the insulin-like growth factor binding domain (IGFBP), Kazal-like serine protease inhibitor domain, trypsin-like serine protease domain, and PDZ-like domain. The protease domain is further divided into three sections: loop D (LD), loop 3 (L3), and not LD or L3 [24,25]. The linker region, located between the Kazal-like and protease domains, was recently described [12,26]. It is theorized that vascular lesions in CARASIL and HTRA1-related CSVD may occur due to a fault in the transforming growth factor (TGF-β) signaling pathway. The TGF-β binding protein, found in the extracellular matrix, is one of the HTRA1 proteolytic substrates. The reduced HTRA1 proteolytic activity due to pathogenic variants in the gene dysregulates the TGF-β signaling inhibition and leads to vasculopathy [27].

Research was completed to evaluate the distribution of the pathogenic variants throughout the protein domains. It was shown that the missense mutations in symptomatic carriers (heterozygotes) tended to cluster around the linker region (amino acid position 166 to 179), which encompasses our in-frame deletion, and LD loop (position 283–286). On the other hand, the missense mutations in CARASIL patients (homozygotes or compound heterozygotes) were rarely found in either the linker or L3/LD domains. The linker and L3/LD regions harbored most of the missense and frameshift variants as well. Both the linker and L3/LD domains are crucial for HTRA1 activation. It is speculated that these differences in the distribution of the HTRA1 variants among CARASIL and HTRA1-related CSVD patients is the reason why the heterozygous parents of CARASIL patients do not usually have symptoms [12]. While the molecular mechanisms of pathogenesis in symptomatic carriers are unclear, prevalent theories include both the dominant-negative effect of mutant alleles, which interfere with the normal function of the wild-type allele [5], and haploinsufficiency as HTRA1 protein expression from the mutant allele is significantly reduced [8,28]. It was observed that mutations with a dominant-negative effect might have a more severe effect on the phenotype and explain the clinical variability of the symptoms in symptomatic carriers [5].

We report a novel HTRA1 heterozygous in-frame deletion. To our knowledge, other in-frame deletion of HTRA1 was reported only twice before in the ClinVar database [29] and medical literature [30]. Furthermore, p.Glu177del is adjacent to our variant; however, no clinical data were provided and the variant is classified as a variant of uncertain clinical significance. On the other hand, pY325_L335del is described as pathogenic, but, unlike our variant, it is located in the protease domain.

We identified heterozygous HTRA1 gene mutation in the proband, but all the living relatives of the patient refused genetic testing. On the other hand, no relatives of the proband had any obvious symptoms of CSVD. Thus, autosomal dominant inheritance cannot be proved in this case. Theoretically, de novo mutation or germline mosaicism in one of the parents is possible although not reported to date [6]. Low grade penetrance in symptomatic carriers of HTRA1 pathogenic variants is known; therefore, the possible phenotypic expression could vary from neuroradiological findings without any clinical symptoms to mild or moderate neurological signs [12]. Thus, the disease may be underdiagnosed, which could be the case in our patient’s family members. In addition, HTRA1-CSVD can be misdiagnosed as sporadic CSVD since aging and arterial hypertension are the contributing factors for both pathologies [3].

Genetic testing for the patient’s siblings and parents would be useful not only for identifying the inheritance pattern and assessment of the recurrence risk but for possible preventive measures too. The usual cardiovascular risk factors are more prevalent in patients with heterozygous rather than with homozygous HTRA1 gene mutations and they contribute to the development of the disease, its progression, and severity. Hence, the control of cardiovascular risk factors is crucial for those who have heterozygous variants of HTRA1 gene mutation [3,6,12].

Our patient was symptomatic and an HTRA1 gene mutation was identified in one allele. Therefore, this case is attributed to HTRA-CSVD or CADASIL2 pathology. HTRA-CSVD usually has a milder course and manifests later compared with CARASIL. Our patient became symptomatic relatively early:—he had a stroke at the age of 36, whereas, typically, acute ischemic events in HTRA1-CSVD patients manifest after the age of 40 [6]. Additionally, the course of the disease was comparably severe as cognitive impairment progressed drastically in a few years. As the patient was a life-long smoker, had arterial hypertension, and high-grade dyslipidemia, it is reasonable to presume that such a severe course could have been avoided with better control of the cardiovascular risk factors [12,31].

The majority of the HTRA1 gene pathogenic variants are described in the Asian population, whereas our patient is European, without known ancestors in Asian countries. It is believed that heterozygous variants could be more frequent and dispersed, although larger studies evaluating the epidemiology of heterozygous HTRA1 carriers are lacking [3].

It is evident that both genetic changes and environmental factors play a role for the phenotype of the heterozygous HTRA1 gene mutations. When the family history is not informative, as in this case, genetic testing should be considered for those patients whose symptoms and extensive changes in brain MRI outbalance the possible impact of cardiovascular risk factors [3].

## Data Availability

Not available.
